# Consumer views on the use of digital tools for reporting adverse drug reactions: a cross-sectional study

**DOI:** 10.1007/s11096-024-01847-2

**Published:** 2024-12-19

**Authors:** Mohammed Gebre Dedefo, Renly Lim, Gizat M. Kassie, Eyob Alemayehu Gebreyohannes, Nava Nikpay Salekdeh, Elizabeth Roughead, Lisa Kalisch Ellett

**Affiliations:** 1 Quality Use of Medicines and Pharmacy Research Centre, UniSA Clinical and Health Sciences, University of South Australia, Adelaide, Australia; 2 UniSA Clinical and Health Sciences, University of South Australia, Adelaide, Australia

**Keywords:** Adverse drug reaction reporting systems, Consumer behavior, Digital technology, Drug-related side effects and adverse reactions, Mobile applications, Pharmacovigilance

## Abstract

**Background:**

The application of digital technologies has shown benefits in enhancing pharmacovigilance activities but consumers views on the use of these tools for this purpose are not well described.

**Aim:**

To explore consumers’ views on using digital tools to report adverse drug reactions (ADRs) and identify key features that consumers want in digital tools for ADR reporting.

**Method:**

An online survey was conducted among adults who had taken medicine in the previous six-months in Australia. The development of questions was guided by the Combined Technology Acceptance Model and Theory of Planned Behaviour (C-TAM-TPB) framework. Responses to closed-ended questions were analysed using descriptive statistics and chi-square/Fisher’s exact test, while free-text responses were analysed using qualitative content analysis.

**Results:**

A total of 494 responses were included in the analysis. Eighty-seven percent of respondents preferred using digital tools for reporting ADRs. Consumers indicated a free-text space for describing ADRs (90%) as important or very important features of digital tools for ADR reporting, followed by acknowledgement of their report submission (87%) and receiving summary of previously reported ADRs (87%). Women (*p* < 0.001), advanced smartphone users (*p* < 0.001), and previous digital healthcare tool users (*p* = 0.017) showed higher intention to use digital tools. Consumers emphasized the importance of ease-of-use, accessibility, receiving medicine safety information, feedback, and advice for reporting ADRs via digital tools.

**Conclusion:**

Consumers prefer using digital tools for reporting ADRs and place high value on features such as a free-text space for describing ADRs, acknowledgement of report submissions, and access to summaries of previously submitted reports.

## Impact statements

- The high consumer intention to use digital tools for ADR reporting could strengthen pharmacovigilance and improve drug safety monitoring.
- Developing an accessible, user-friendly ADR reporting tool that provides feedback and medicine safety information could potentially increase reporting rates and enable consumers to play an active role in medication safety.
- Addressing consumer concerns regarding data reliability, overreporting, and privacy in digital ADR reporting tools is imperative to establish trust and ensure data accuracy, thereby improving rate of ADR reporting and public health outcomes.

## Introduction

Adverse drug reactions (ADRs) are defined as “any response to a drug which is noxious and unintended, and which occurs at doses normally used for prophylaxis, diagnosis, or therapy of disease, or the modification of physiological function” [1]. ADRs are associated with poor quality of life, hospitalisation, and increased healthcare costs [2–4]. Spontaneous reporting is one mechanism to identify ADRs, with potential flow on effects of reducing patient harm [5–8]. It enables regulatory authorities to monitor the safety of medicines and implement timely interventions when necessary [5, 6]. However, studies have shown there is underreporting of ADRs to regulators, particularly by consumers [9–11].

Systematic reviews identified unawareness, complacency, lethargy, limited knowledge of ADR self-reporting, and not perceiving reporting as their responsibility as the main reasons for underreporting by consumers [12, 13]. Initially, ADR reporting used paper-based forms, which were time-consuming, had poor recording quality, and limited accessibility [12, 14, 15]. To overcome these issues and improve ADR reporting, new technologies like mobile apps and web-based systems have been implemented [14, 15]. These digital tools offer simplicity, ease of use, broad accessibility, direct data transfer to regulatory authorities, cost-effectiveness, improve user engagement through two-way communication and updates on medicine safety [16, 17]. However, limitations include insufficient data collection needing supplementary reporting, challenges in areas with poor internet connectivity, the inability to save or share submitted reports, and data security concerns [16–18].

International studies have indicated that the use of digital tools can improve the reporting of ADRs [16, 19]. A study that evaluated the impact of 16 mobile apps found that ADR reporting increased by a range of 12% to 597% for thirteen of the apps in the first 12-months of the app’s launch [16]. Another study found that web-based ADR reporting increased ADR reporting to 13,515 reports compared to 4832 traditionally submitted reports in Germany in 2019 [20]. However, both studies reported only the total number of increased reports following launch of the apps/website and did not specify what proportion of reports were submitted by consumers, so, it remains unclear whether the apps successfully increased consumer reporting of ADRs.

Different countries around the world use a variety of methods for ADR reporting. Australia is a country with a long-standing history of ADR reporting by consumers, first established in 1964 at the inception of its spontaneous reporting system [21]. The national medicines regulator, the Therapeutic Goods Administration (TGA) recommends consumers report any suspected ADRs associated with medicines [22]. Consumers can report ADRs by telephone, the TGA website, email or using a paper-based form (the ‘blue card’). The TGA’s online reporting system facilitates the post-market monitoring of the safety of therapeutic goods’, and is designed to ensure the confidentiality of personal information [23]. TGA provides consumers with confirmation upon submission of their reports, which could enhance trust and encourage future reporting [24]. However, a survey conducted in 2023 revealed only 12% of consumers use the TGA’s online ADR reporting platform [25], indicating potential barriers in accessibility or awareness.

Using electronic tools for ADR reporting, and in particular app-based tools, is a relatively new concept for consumers [26]. Various theoretical models have been developed to understand the behavioural intentions of consumers towards the acceptance of new technology. One such model is the Combined Technology Acceptance Model and Theory of Planned Behaviour (C-TAM-TPB) [27]. This theory integrates the cognitive approach of the TPB which focuses on individuals' attitudes and beliefs toward new technology, and the TAM which assumes that users decide when and how they will use a new technology based on its perceived usefulness and ease-of-use [28]. The C-TAM-TPB framework is useful when aiming to understand the behavioral intentions of consumers towards the acceptance of new technology, because it provides a comprehensive framework for understanding the factors related to attitudes, beliefs, and the use of technology that affect consumers’ behaviour when adopting new technology.

### Aim

This study investigated consumers’ views on using digital tools to report ADRs and identified the features that consumers consider important in digital tools to report ADRs, using the C-TAM-TPB as a framework among adults in Australia.

### Ethics approval

Ethics approval was obtained from the University of South Australia Human Research Ethics Committee on May 4, 2023 (protocol number: 205183). This study adheres to the Australian National Statement on Ethical Conduct in Human Research (updated 2023) and the Declaration of Helsinki. All participants provided informed consent prior to participating in the study.

## Method

### Study design

A cross-sectional survey was conducted using Qualtrics [29]. The survey was open to adults aged 18 or older living in Australia who had self-reported taking medicines within the previous six months.

### Questionnaire development, recruitment strategy and data collection

A structured questionnaire was developed through a review of prior literature on the topic [16, 30–35]. The questionnaire comprised 27 items and was divided into five sections: (i) general questions about the participant’s experience with medicine use (four items), (ii) knowledge of ADR reporting (six items), (iii) practices of ADR reporting (six items), (iv) views on the use of digital tools for ADR reporting (seven items), and (v) consumer’s socio-demographic characteristics (four items).

The survey was conducted online. Although we offered paper-based copies of the survey for participants who preferred this method, none chose this option. The study utilised a convenience sampling technique to recruit participants. To facilitate access to the survey, a flyer was designed which provided information about the study objectives and included a quick response (QR) code and survey link. The flyer was advertised on social media platforms, such as Twitter and Facebook, and displayed in community pharmacies, hospital pharmacies, and general practitioner (GP) clinic waiting areas. Additionally, the Pharmaceutical Society of Australia (PSA) assisted in distributing the flyer to community pharmacies. More details on the survey design, recruitment strategy, data collection and findings on consumers’ knowledge and practices on ADR reporting have been reported previously [25]. In this paper, we present results relating to the perceptions of consumers regarding important features of digital tools for ADR reporting, and their views on the use of digital tools for ADR reporting.

Consumers’ perception of the importance of the features of digital tools for reporting ADRs were measured using eight statements on a five-point Likert scale of “not at all important,” “somewhat important,” “important,” “very important,” and “don’t know.” Consumers also had the opportunity to provide free text responses.

The six constructs of C-TAM-TPB framework were used to assess consumers’ views on the use of digital tools for ADR reporting [27]. Nine statements were included, and consumers were asked to indicate their agreement using a five-point Likert scale (“strongly disagree,” “disagree,” “neutral,” “agree”, “strongly agree”). Two statements related to the construct of perceived usefulness, one statement on ease of use, one statement on attitude, two statements on subjective norm, two statements on perceived behavioural control and one statement on behavioural intention. Survey data was collected between May and September 2023.

### Data analysis

This study employed a mixed (both quantitative and qualitative) method of analysis.

### Quantitative analysis

Responses from 494 participants who responded to all survey questions relating to consumers’ views on the use of digital tools were included. Participants had to answer all questions relating to their views on the use of digital tools in order to be included in the analysis, but did not have to provide responses to all questions relating to consumer perceptions of the importance of digital tool features and views on the use of digital tools for reporting ADR in order to be included. Therefore, the denominators for each question in the sections relating to importance of digital features and consumers’ views of digital tool use vary according to the total number of responses to that question.

Descriptive statistics were used to describe the characteristics of survey participants. Chi-square test or Fisher’s exact test (as appropriate) were conducted to determine whether there were significant differences in consumers’ characteristics and intention to use digital tools for reporting ADRs. To determine whether there were significant differences between consumer’s characteristics and behavioural intention to use digital tools to report ADRs, the Likert scale categories were dichotomised, with strongly disagree/disagree/neutral responses grouped together for analysis and agree/strongly agree responses grouped for analysis. The internal consistency of Likert scale responses was assessed using Cronbach’s alpha. The Cronbach’s alpha coefficient for items measuring consumers’ perceptions of the importance of the features of digital tools for reporting ADRs was 0.80, while the Cronbach’s alpha coefficient for consumers’ views on using digital tools for ADR reporting, based on the C-TAM-TPB framework, was 0.86, indicating a high internal consistency [36]. SPSS version 28 (IBM Corporation, New York, USA) was used for data analysis. A p-value less than 0.05 was considered statistically significant.

### Qualitative analysis

Responses from ninety-nine consumers who provided free-text responses were included in the qualitative content analysis [37]. An inductive approach was used during the analysis process, where coding and category development were derived from the free-text responses [38]. The developed categories were then mapped into the themes of the TAM [39].

NVivo 14 software (QSR International Pty Ltd., Doncaster, Victoria, Australia) was utilised. Two researchers (MGD and NNS) familiarised themselves with free-text responses, independently generated codes, and then discussed the codes to reach a consensus. A third researcher (EAG) resolved disagreements. Categories were developed for related codes by two researchers (MGD and NNS), which were then reviewed and cross-checked with the research topic and question to ensure the identified categories related to the aims of the study. The categories were subsequently reviewed, discussed, and consensus was reached among all researchers. The categories were then mapped into the themes of TAM [39].

## Results

### Quantitative analysis

#### Sociodemographic characteristics

Three quarters of participants were women, and 69% were aged under 65. Most respondents had a high level of education, with 36% having a bachelor’s degree as their highest qualification and 34% holding a postgraduate degree. Almost all participants (94%) reported that they use digital tools for healthcare activities (Table 1).

**Table 1:** Characteristics of the respondents and their experience with medicine use and digital tool use (N = 494)

Characteristics of the respondents		Number (%)
Gender	Man	113 (22.9%)
	Woman	370 (74.9%)
	Other gender or no response	11 (2.2%)
Age	18–44	160 (32.4%)
	45–64	180 (36.4%)
	65 or older	153 (31.0%)
	No response	1 (0.2%)
Highest level of education	Secondary school	88 (17.8%)
	VET^a^ certificates, diploma, associate diploma, associate degree or graduate diploma	36 (7.3%)
	Bachelor’s degree	176 (35.6%)
	Postgraduate degree	170 (34.4%)
	Other	8 (1.6%)
	No response	16 (3.2%)
The average number of medicines taken in the past 6 months	1–4	282 (57.1%)
	≥ 5	165 (33.4%)
	No response	47 (9.5%)
Did you start taking a new medicine for the first time in the past 6 months?	Yes	244 (49.4%)
	No	246 (49.8%)
	Not sure	4 (0.8%)
Have you ever used digital tools for healthcare activities?	Yes	466 (94.3%)
	No	27 (5.5%)
	No response	1 (0.2%)
Level of experience with using a smartphone	Limited (I use it to make a phone call and send text messages only)/do not use a smartphone	19 (3.8%)
	Moderate (I use it for online searches such as on Google and Safari)	89 (18.0%)
	Advanced (I use it to make e-payments, book appointments using apps etc.)	377 (76.3%)
	No response	9 (1.8%)

^a^VET,  Vocational Education and Training

#### Consumers’ perception of the importance of the features of digital tools for ADR reporting

**Fig. 1:**
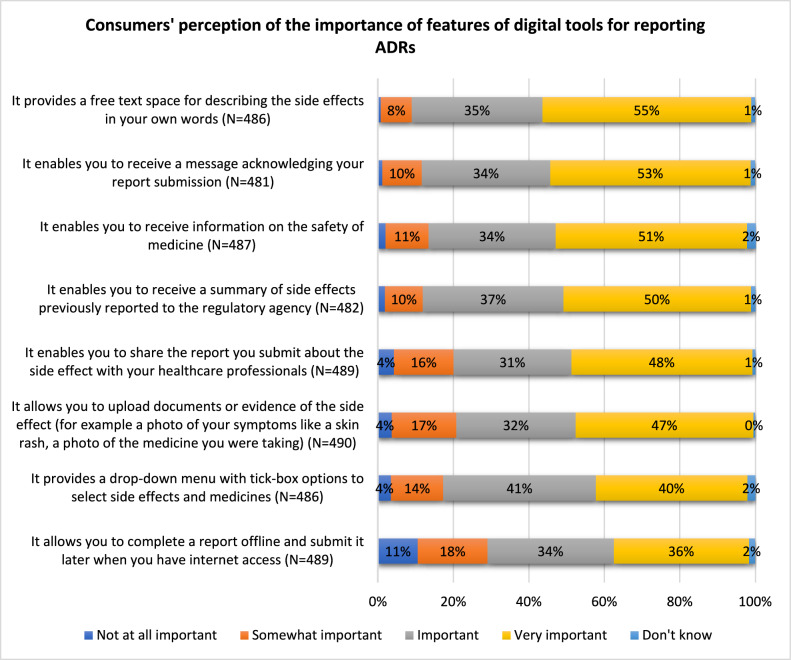
Consumers' perception of the importance of features of digital tools for reporting ADRs

Ninety percent (n = 437/486) of respondents reported that it is important or very important for a digital ADR reporting tool to provide a free text space that allows consumers to describe ADRs in their own words. Similarly, 87% (n = 419/481) of respondents said that it was important or very important for a digital ADR reporting tool to acknowledge report submission (Fig. 1).

#### The views of consumers on the use of digital tools for reporting ADRs

**Fig. 2:**
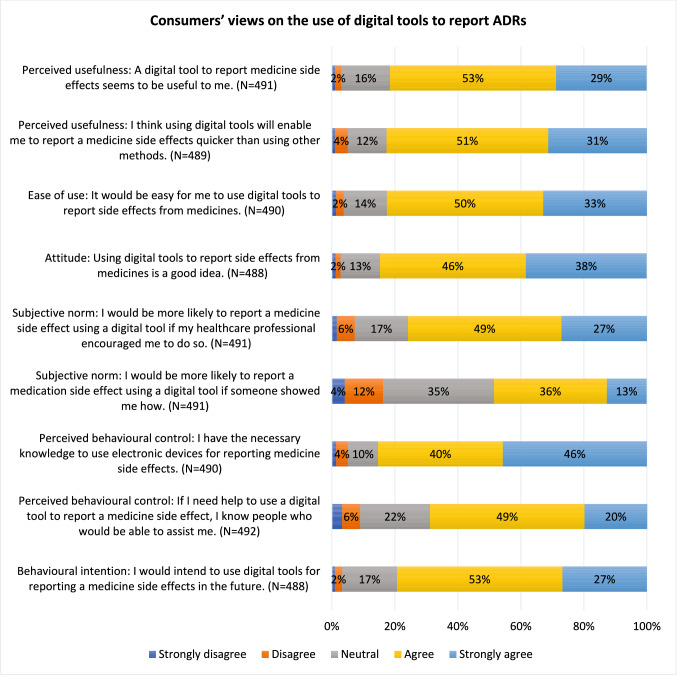
Consumers’ views on the use of digital tools to report ADRs

Among the participants who responded to questions related to the C-TAM-TPB constructs, more than 80% agreed/strongly agreed with the statements regarding perceived usefulness, ease of use, attitude, behavioural intention and one of the two statements on perceived behavioural control (Fig. 2). For example, 82% (n = 400/491) agreed/strongly agreed that a digital tool for reporting ADRs would be useful, and 82% (n = 404/489) agreed/strongly agreed that it would likely allow for faster reporting compared to other methods (Fig. 2).

#### Statistical difference between behavioural intention to use digital tools to report ADRs and respondent characteristics.

There were statistically significant differences in gender, experience of using digital tools for healthcare activities, and level of experience with smartphones between consumers who agreed/strongly agreed compared to those who strongly disagreed/disagreed/were neutral to the question relating to behavioural intention to use digital tools. More women (83%) than men (67%) (*p* < 0.001) agreed/strongly agreed about the future use of digital tools for ADR reporting. Similarly, respondents who used digital tools for healthcare activities (81%) agreed/strongly agreed compared to those who did not (58%) (*p* = 0.017). Furthermore, those with advanced smartphone experience (83%) agreed/strongly agreed than those with moderate (73%) or limited/no experience (47%) (*p* < 0.001) (Table 2).

**Table 2:** Characteristics of the respondents by intention to use digital tools to report ADRs in the future (N = 488)

Variables		Intention to use digital tools to report ADRs in the future		*p*-value
		Strongly disagree/ disagree/neutral, n = 101	Agree/strongly agree, n = 387	
Gender	Man	36 (33.0%)	73 (67.0%)	< 0.001
	Woman	62 (16.8%)	307 (83.2%)	
	Others/no response	3 (30.0%)	7 (70.0%)	
Age	18–44	32 (20.5%)	124 (79.5%)	0.952
	45–64	39 (21.7%)	141 (78.3%)	
	65 or older	30 (19.9%)	121 (80.1%)	
	No response	0 (0.0%)	1 (100%)	
Highest level of education	Secondary school	13 (15.3%)	72 (84.7%)	0.642
	VET certificates, diploma, associate diploma, associate degree or graduate diploma	6 (16.7%)	30 (83.3%)	
	Bachelor’s degree	39 (22.3%)	136 (77.7%)	
	Postgraduate degree	38 (22.6%)	130 (77.4%)	
	Others/no response	5 (20.8%)	19 (79.2%)	
The average number of medicines taken	1–4	52 (18.7%)	226 (81.3%)	0.296
	≥ 5	36 (22.0%)	128 (78.0%)	
	No response	13 (28.3%)	33 (71.7%)	
Start taking a new medicine for the first time	Yes	45 (18.5%)	198 (81.5%)	0.237
	No/not sure	56 (22.9%)	189 (77.1%)	
Used digital tools for healthcare activities	Yes	90 (19.5%)	371 (80.5%)	0.017
	No	11 (42.3%)	15 (57.7%)	
	No response	0 (0.0%)	1 (100.0%)	
Level of experience with using a smartphone	Limited/do not use a smartphone	10 (52.6%)	9 (47.4%)	< 0.001
	Moderate	24 (27.3%)	64 (72.7%)	
	Advanced	64 (17.1%)	310 (82.9%)	
	No response	3 (42.9%)	4 (57.1%)	

### Ranking of ADR reporting methods

When provided a list of ADR reporting methods and asked to rank them in order of their preference of use, with the option to assign equal rankings, 49% (n = 227/468) of respondents reported that using a mobile app would be their preferred way to report ADRs and 38% (n = 177/468) reported that a website would be their most preferred way to report ADRs. Only 20% (n = 92/468) of respondents reported that their preferred method of ADR reporting was via a paper-based form, and 20% (n = 93/468) indicated a preference for reporting over the phone.

Chi-squared tests showed that there was a significant difference between consumers' age and their preferences for reporting ADRs. A higher proportion of consumers aged 18–44 years listed a website/web app as their first choice than consumers aged 45 years or older (*p* = 0.038, Table 3). A higher proportion of consumers aged 65 or older listed a phone conversation as their first choice compared to younger consumers (*p* = 0.015, Table 3).

**Table 3:** Respondents' preference of ADR reporting methods by their age (N = 467)

Reporting methods	Rank of preference	Age of respondents			*p-value*
		18–44 (n = 152)	45–64 (n = 172)	65 or older (n = 143)	
Mobile app	1st choice	77 (50.7%)	83 (48.3%)	67 (46.9%)	0.451
	2nd choice	44 (28.9%)	45 (26.2%)	41 (28.7%)	
	3rd choice	21 (13.8%)	19 (11.0%)	19 (13.3%)	
	4th choice	10 (6.6%)	25 (14.5%)	16 (11.2%)	
Website or web app	1st choice	66 (43.4%)	57 (33.1%)	53 (37.1%)	0.038
	2nd choice	65 (42.8%)	80 (46.5%)	49 (34.3%)	
	3rd choice	14 (9.2%)	22 (12.8%)	28 (19.6%)	
	4th choice	7 (4.6%)	13 (7.6%)	13 (9.1%)	
Phone conversation	1st choice	18 (11.8%)	39 (22.7%)	34 (23.8%)	0.015
	2nd choice	27 (17.8%)	17 (9.9%)	28 (19.6%)	
	3rd choice	52 (34.2%)	60 (34.9%)	36 (25.2%)	
	4th choice	55 (36.2%)	56 (32.6%)	45 (31.5%)	
Paper-based form	1st choice	21 (13.8%)	34 (19.8%)	36 (25.2%)	0.311
	2nd choice	15 (9.9%)	16 (9.3%)	15 (10.5%)	
	3rd choice	48 (31.6%)	54 (31.4%)	43 (30.1%)	
	4th choice	68 (44.7%)	68 (39.5%)	49 (34.3%)	

### Qualitative content analysis

Ninety-nine consumers provided free-text responses regarding what they want from digital tools for ADR reporting. Analysis of these responses generated 133 concepts resulting in 43 codes, which were subsequently categorised into nine categories based on their conceptual similarity. The categories were: accessibility, ease of use, provision of feedback, medicine safety information, provision of guidance and support for users, other digital tool functions, data privacy and security, reliability of report, and opinions on importance of digital tools. These categories were subsequently mapped into three themes of the TAM, including perceived ease of use, perceived usefulness, and attitude.

### Perceived ease of use

Two categories were classified under perceived ease of use: accessibility and ease of use.

#### Accessibility

Five comments were made by respondents about the accessibility of digital tools for consumers. The respondents emphasized the need to be able to access digital tools on various platforms, as well as different operating systems to facilitate the process of reporting ADRs. Additionally, making it accessible to individuals with disabilities and implementing colours that are suitable for individuals with colour blindness.

*“A digital tool needs to be clear and well set out. It also needs to be able to be accessed via computer and not just via a smartphone or tablet to assist those who need a bigger screen.”(P17)*

#### Ease of use

Respondents made twelve comments regarding the ease of use of digital tools. They suggested making digital tools user-friendly, simple, easy to navigate, report without creating account, and usable for consumers with limited experience with technology. Respondents also wanted digital tools to use plain English, incorporating generic drug names and having a quick page load speed.

*“I think this sounds like a wonderful idea, but it occurs to me that the UI [User Interface] would need to be super simple, just because there may be users who are not technologically savvy.”(P38)*

### Perceived usefulness

Four categories were classified under perceived usefulness: feedback, medicine safety information, providing guidance and support for users and other digital tool functions.

#### Feedback

Fourteen comments were provided by respondents suggesting that an acknowledgement of submission of their report and feedback on the progress of their report would enhance utilisation of digital tools to report ADRs.

*“I would also like to receive some information about what will happen with my report, if I can expect a response.”(P43)*

#### Medicine safety information

A total of thirty comments were received from consumers regarding the significance of obtaining medicine safety information through digital tools. Additionally, there was interest in linking digital tools to existing medication management applications.

*“Enables me to obtain information, including possible side-effects, about the medication.”(P30)*

Consumers said that they want a digital reporting tool that includes a feature allowing them to access information on previous ADR reports for medicines.

*“Would be good to see the percentage of others who reported similar side effects.”(P98)*

#### Providing guidance and support for users

Thirty-nine comments were made suggesting the importance of receiving advice on whether consumers need to consult their healthcare professionals (HCPs) or visit the emergency department and a feature that provides a reminder to consult HCPs.

*“Gives advice on whether I should cease the medication and/or seek further advice from my doctor about the side effects.”(P33)*

Consumers suggested that digital tools for ADR reporting should gather detailed information on how consumers handle their medicines, other chronic conditions, co-ingested food and drinks, supplements, as well as information on other medicines that were taken and the reason for taking them. Consumers emphasized the importance of obtaining contact information for HCPs and regulatory agencies that could assist them.

*“Asking about how the medicine was handled (eg some people take the tablets out of the blister pack and put it into a plastic container divided into days. This may affect side effects?”(P21)*

#### Other digital tool functions

Nine comments were made by consumers suggesting the addition of functionalities such as product scanning and photo uploading, keeping a record of submissions or emailing a copy to the individual making the report, the ability to report ADRs experienced by someone else, noise recording for breathing or type of cough, and the option to copy the report to a general practitioner to facilitate reporting of ADR using digital tools.

*“Cc [carbon copy] to my G.P. [general practitioner] Too.”(P23)*

*“Scan the product upload a photo.”(P79)*

### Attitude

Three categories were classified under attitude: Data privacy and security, reliability of report and opinions on importance of digital tools.

#### Data privacy and security

Four comments were made on the importance of safeguarding the privacy and security of personal information when reporting ADRs. Consumers suggested the need for assurance regarding the handling of their information, e.g. receiving a code via mobile phone to log onto the site for security.

*“I would like to know unequivocally that my privacy was not at risk.”(P46)*

#### Reliability of reports

Nine comments were made on concerns with the reliability of ADR reports by consumers. They suggested that consumers should upload evidence of their medicine and ADRs, and digital tools should be equipped with features that prompt for corrections in the event of mistakes made during the reporting process. Respondents were also concerned about false reporting and over reporting with digital tools.

*“I would be concerned that this feature could be used by some people to over report adverse reactions and affect the public perception of a particular medication.”(P42)*

#### Opinions on importance of digital tools

Eleven comments were categorised under opinions on importance of digital tools. Participants highlighted that they feel it is important to have digital tools available to report ADRs, as they are likely to be convenient to use. They suggested using digital tools to report ADRs directly to regulatory agencies may help bypass HCPs if they are dismissive of a patients’ ADR concerns or who do not realise that a symptom reported by a patient may be an ADR.

*“At times I have had my concerns about side effects dismissed by health professionals as not related to the medications or not significant enough to be concerned about, which may well be true. However having a place to describe them and be noted would be valuable as it is possible many people are having there [sic] concerns dismissed about the same medications and these effects are not being recorded.”(P19)*

However, consumers also suggested involvement of HCPs during reporting is helpful to avoid errors.

*“In the hands of genuine healthcare professionals, it would be helpful.”(P69)*

## Discussion

This study assessed consumers’ views on the use of digital tools to report ADRs and identified their perceptions of the important functions of digital tools for ADR reporting. Most consumers in this survey (87%) preferred digital tools for reporting ADRs to paper-based forms and phone conversations. A free text space for describing ADRs in their own words (90%) was the top feature indicated by consumers as being important, followed by a digital feature acknowledging their report submission (87%), and receiving a summary of previously submitted ADRs (87%).

Our study found that consumers have concerns about the privacy and security of their personal information, the complexity of digital tools, the reporting of false data, and over-reporting of ADRs using digital tools. Our findings are in line with those of a previous study conducted in Ghana that highlighted concerns about mobile app use for ADR reporting, including the privacy and security of personal information, as well as the ease of understanding the information provided within the app [40]. A study conducted in Japan identified several barriers to using online web-based ADR reporting, including having too many sections to fill out, lack of familiarity with web operations, unfamiliar terminology in the online form, and the absence of feedback [41]. While digital tools have a role in improving the rate of ADR reporting [16, 42], our findings indicate the need for addressing consumers’ concerns regarding privacy, security, and the complexity of digital tools.

This study showed that consumers want to receive feedback and advice regarding ADRs, as well as being able to access safety information and previous ADR reports on the medicine. Incorporating these elements may facilitate the use of digital tools for reporting ADRs. Studies conducted in Europe and Japan also reported that patients would like to receive feedback on their report [41, 43] and an overview of ADRs previously reported by others[32]. These findings highlight the importance of integrating feedback mechanisms and expert support, developing user-friendly digital tools, and providing medicine safety information within digital reporting platforms to enhance consumer engagement and improve ADR reporting rates and medication safety.

Survey respondents perceived digital tools as useful for reporting ADRs, making the process of reporting quicker than other methods. They believe that using digital tools is likely to be an easy process, and they would be more likely to report ADRs using digital tools if HCPs encouraged them to do so. In a study conducted in Italy, researchers evaluated the usability of a mobile app for monitoring ADRs during the COVID-19 pandemic and found that various elements, including ease of use, accessibility across multiple platforms, anonymity of participants, data protection, and support from healthcare workers were important contributors [44]. These insights highlight the need to develop a digital tool for consumers that is easy to use, quick to complete, and recommended to consumers by HCPs to increase interest in using digital reporting tools and improve the rate of ADR reporting.

In this study, 87% of consumers preferred digital tools for reporting ADRs. European studies also show consumer interest in mobile apps for ADR reporting ranges from 38 to 56% [32, 45]. Our findings revealed that younger individuals were more likely to use digital tools for reporting ADRs than older individuals, aligning with the European study showing younger patients’ greater interest in apps for ADR reporting and drug safety information [32]. These studies highlight the high interest of consumers in digital ADR reporting tools, which could potentially lead to an increase in ADR reporting.

### Study strengths and future implications

The study’s strengths lie in the use of diverse recruitment methods and a comprehensive questionnaire integrating multiple-choice, Likert scale, and free-text responses, enabling both quantitative and qualitative analyses. This approach uncovered additional consumer views on digital tools and preferences beyond predefined survey options, highlighting the importance of user-friendly design and ease of use. The findings emphasise the importance of understanding consumer preferences regarding digital tools for ADR reporting to develop user-friendly platforms. Our findings indicate high consumer interest in digital tools and emphasize important features to include. Future research should assess consumer perceptions, identify barriers to existing digital tools, and evaluate what features enhance ADR reporting. These insights are important for developing improved, user-friendly tools that meet consumer needs and improve ADR reporting.

### Study limitations

The convenience sampling method employed may have introduced selection bias, as it is likely that individuals who were already inclined to report ADRs or were comfortable using digital tools were more likely to participate. In addition, the convenience sampling method used means that it is not possible to determine the total number of possible respondents to the survey. The online nature of the study is likely to have led to a lack of representation for those who do not use electronic devices. Compared to the general population of Australian adults, where 51% of people have a bachelor’s degree or higher [46, 47], a larger proportion of participants in our study held bachelor's degrees or higher (70% of study participants). Additionally, a higher proportion of women (75% vs 50%) and individuals aged 65 years or older (31% vs 17%) participated [48–50] compared to the general Australian population. These factors may limit the generalisability of the study’s findings and our results should be interpreted with these limitations in mind.

## Conclusion

Digital tools were preferred to other methods for ADR reporting by most consumers who completed this survey. Women, those familiar with smartphone activities like e-payments and booking appointments, and those who had used digital tools for healthcare activities reported higher intentions to use digital tools for ADR reporting in the future. Consumers emphasized the importance of ease of use, accessibility, receiving medicine safety information, feedback, and advice for reporting ADRs via digital tools. Consumers also expressed their concerns regarding the reliability of reports and the privacy and security of personal information when using digital tools for reporting ADRs that need to be addressed. Future research should focus on whether tools that incorporate these features lead to higher rates of ADR reporting by consumers.
